# Nanoscale proximity of a free interface and an ice nucleating particle is a sufficient condition for contact freezing

**DOI:** 10.1038/s42004-021-00477-3

**Published:** 2021-03-15

**Authors:** Teresa S. Ortner

**Affiliations:** Communications Chemistry, https://www.nature.com/commschem

## Abstract

Contact freezing of water is a very fast and common process that is still not well understood due to challenges in probing this microscopic phenomenon. Now, molecular dynamics simulations help to explain experimental data of contact freezing, showing a connection between water’s suspected propensity to undergo surface freezing and the kinetic enhancement during contact nucleation.

During atmospheric ice nucleation, dry ice nucleating particles and a water droplet can collide, resulting in accelerated nucleation. This complex process is called contact freezing and occurs within picoseconds and at the nanoscale. Its kinetics and mechanism are affected by many factors, which have yet to be fully understood. Now, Sarwar Hussain and Amir Haji-Akbari from Yale University, USA, simulated the heterogeneous nucleation of two water model ices within supported nanofilms of the supercooled liquid, and found a connection between water surface freezing propensity and kinetic enhancement during contact nucleation (10.1021/jacs.0c10663)^1^.

“It has been known since the 1950s that contact freezing occurs at rates several orders of magnitude higher than immersion freezing, which is the dominant mode of heterogeneous ice nucleation”, says Haji-Akbari. “The origin of this enhancement, however, is still a mystery, with at least six competing theories for it in the current literature”. Collision-induced transient perturbations have long been thought to be the main cause of the fast freezing, but experimental findings^2^ indicated the importance of nucleating particles being in close proximity to free liquid interfaces.

To isolate the governing mechanistic factors, Hussain and Haji-Akbari used molecular dynamics simulations alongside their recently developed forward flux sampling algorithm with jumpy order parameters^3^ on two water-like tetrahedral liquids. “Midway through the study, we figured out that some of our calculations were strongly impacted by finite size effects. As a result, we repeated those calculations at a lower temperature and within considerably larger simulation boxes”, recalls Haji-Akbari. To detect and treat such finite size artifacts in studies of heterogeneous ice nucleation, the researchers have developed a rigorous heuristic^4^. Analyzing the results using a generalization of classical nucleation theory, the researchers ultimately found that nanoscale proximity causes freezing, but only if the free interface has a tendency to enhance homogeneous nucleation. That tendency is suspected for water and is referred to as surface freezing propensity^5^.

In addition, a new nucleation mechanism was identified that involves the formation of hourglass-shaped, crystalline nuclei that grow at both the free interface within the supercooled water and from the interface with the support (Fig. 1a). Hourglass-shaped nuclei are easier to form, as the free interface of a water microdroplet is suspected of enhancing ice nucleation in the absence of other ice nucleating particles, and will likely do the same for heterogeneous nucleation if the ice nucleating surface is sufficiently close to it. That effect alone was, however, not enough to quantitatively explain the observed additional increase in the freezing rate over classical heterogeneous nucleation. The researchers found a modulation of the free interfacial structure of the simulated water films, which results in a decrease in the effective contact angle.

“Our work provides a framework for understanding crystal nucleation in supported nanofilms or in liquids that are next to surfaces with nano-patches of different chemistries and topographies. Examples of such surfaces include self-assembled block copolymers, and ice-nucleating and antifreeze proteins,” concludes Haji-Akbari.

**Fig. 1:**
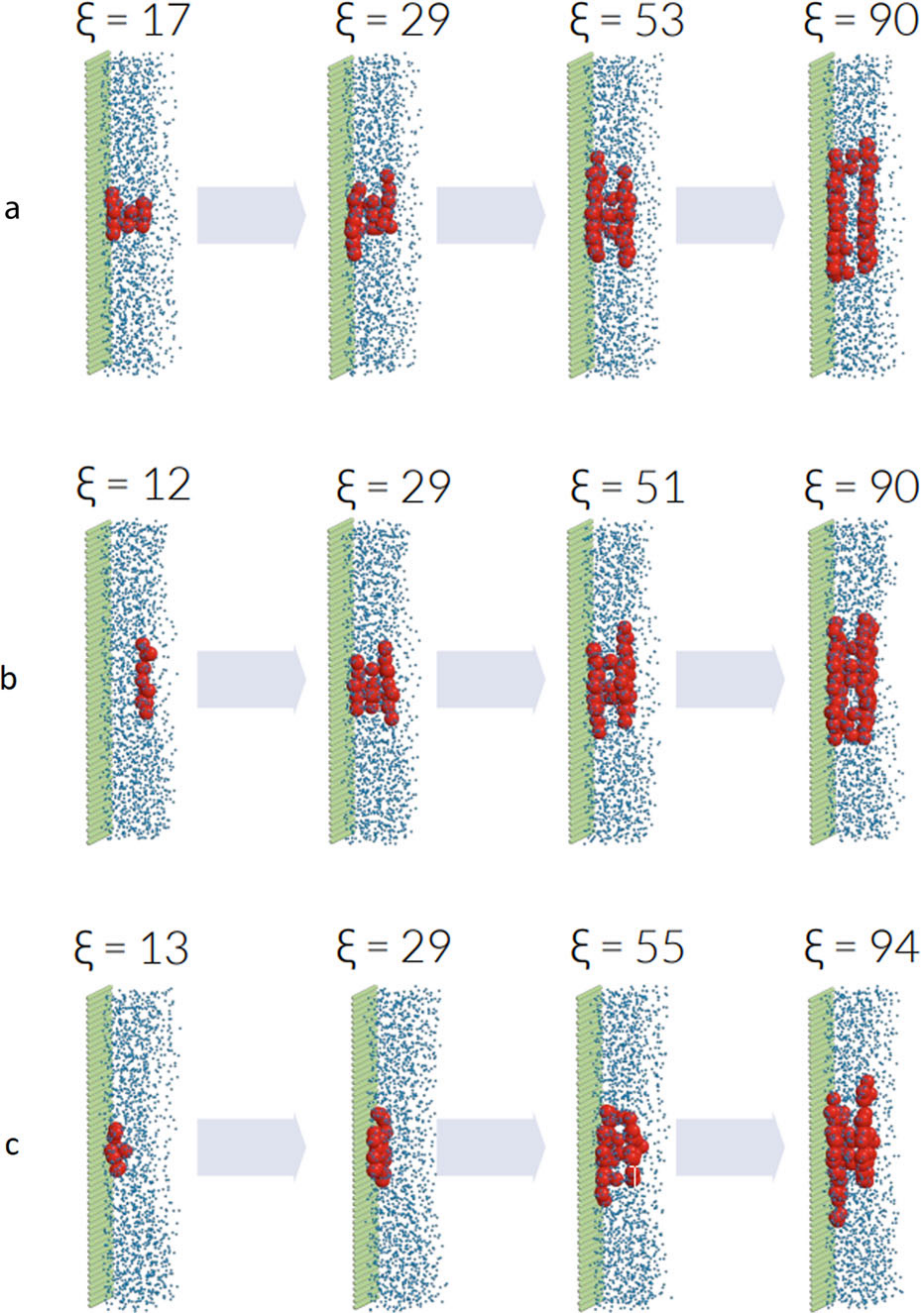
Nucleation pathways in an ultrathin film of model water at 170 K. Three representative freezing pathways for nuclei that start (**a**) at both interfaces, (**b**) at the free interface only, and (**c**) at the ice nucleating particle (here the graphene support). The ice nucleating particles and water droplets are depicted in dark red and light blue, respectively, on a graphene support (green). The number of molecules within the largest crystalline nucleus is given as the order parameter ξ. Each configuration is a progeny of the configuration to its left. Reproduced from^1^, copyright (2021) American Chemical Society.
